# Modelling the impact of local reactive school closures on critical care provision during an influenza pandemic

**DOI:** 10.1098/rspb.2010.2688

**Published:** 2011-02-02

**Authors:** Thomas House, Marc Baguelin, Albert Jan Van Hoek, Peter J. White, Zia Sadique, Ken Eames, Jonathan M. Read, Niel Hens, Alessia Melegaro, W. John Edmunds, Matt J. Keeling

**Affiliations:** 1 Warwick Mathematics Institute, University of Warwick, Gibbet Hill Road, Coventry CV4 7AL, UK; 2 School of Life Sciences, University of Warwick, Gibbet Hill Road, Coventry CV4 7AL, UK; 3 Immunisation Department, Health Protection Agency, 61 Colindale Avenue, London NW9 5EQ, UK; 4 Modelling and Economics Unit, Health Protection Agency, 61 Colindale Avenue, London NW9 5EQ, UK; 5 MRC Centre for Outbreak Analysis and Modelling, Department of Infectious Disease Epidemiology, Imperial College Faculty of Medicine, Norfolk Place, London W2 1PG, UK; 6 Health Services Research Unit, London School of Hygiene and Tropical Medicine, Keppel Street, London WC1E 7HT, UK; 7 Infectious Diseases Epidemiology Unit, London School of Hygiene and Tropical Medicine, Keppel Street, London WC1E 7HT, UK; 8 Institute of Infection and Global Health, University of Liverpool, Leahurst Campus, Neston CH64 7TE, UK; 9 Interuniversity Institute for Biostatistics and Statistical Bioinformatics, Hasselt University, Agoralaan 1, B3590 Diepenbeek, Belgium; 10 Centre for Health Economics Research and Modelling Infectious Diseases and Centre for the Evaluation of Vaccination, Vaccine and Infectious Disease Institute, University of Antwerp, Belgium; 11 DONDENA Centre for Research on Social Dynamics, Bocconi University, Via Guglielmo Röntgen n.1, 20136 Milan, Italy

**Keywords:** influenza, pandemic, schools, modelling, intensive care unit, hospitals

## Abstract

Despite the fact that the 2009 H1N1 pandemic influenza strain was less severe than had been feared, both seasonal epidemics of influenza-like-illness and future influenza pandemics have the potential to place a serious burden on health services. The closure of schools has been postulated as a means of reducing transmission between children and hence reducing the number of cases at the peak of an epidemic; this is supported by the marked reduction in cases during school holidays observed across the world during the 2009 pandemic. However, a national policy of long-duration school closures could have severe economic costs. Reactive short-duration closure of schools in regions where health services are close to capacity offers a potential compromise, but it is unclear over what spatial scale and time frame closures would need to be made to be effective. Here, using detailed geographical information for England, we assess how localized school closures could alleviate the burden on hospital intensive care units (ICUs) that are reaching capacity. We show that, for a range of epidemiologically plausible assumptions, considerable local coordination of school closures is needed to achieve a substantial reduction in the number of hospitals where capacity is exceeded at the peak of the epidemic. The heterogeneity in demand per hospital ICU bed means that even widespread school closures are unlikely to have an impact on whether demand will exceed capacity for many hospitals. These results support the UK decision not to use localized school closures as a control mechanism, but have far wider international public-health implications. The spatial heterogeneities in both population density and hospital capacity that give rise to our results exist in many developed countries, while our model assumptions are sufficiently general to cover a wide range of pathogens. This leads us to believe that when a pandemic has severe implications for ICU capacity, only widespread school closures (with their associated costs and organizational challenges) are sufficient to mitigate the burden on the worst-affected hospitals.

## 1. Introduction

The 2009 outbreak of H1N1 influenza was first reported in Mexico in April, and initial reports suggested that it was associated with a high level of case fatalities [1]. On 28 April, the first cases were confirmed in the UK, and on 11 June the World Health Organization officially declared a pandemic. Data from a variety of countries in both the Northern and Southern Hemispheres have since shown that this strain was associated with far lower mortality than first anticipated. Incidence was highest in children—the elderly, who usually suffer the most adverse reactions to influenza, appear to have had a much lower infection risk [2]. However, despite the fact that this infection was relatively mild, especially in comparison with previous influenza pandemics (particularly the 1918 outbreak), there were still substantial concerns over the probable burden upon health-care services. Intensive care units (ICUs) are expected to come under particular pressure near the peak of any novel epidemic, which has implications for the care of those most severely affected. There are two options to alleviate this burden: either increasing ICU capacity or decreasing the epidemic peak. During the latter part of 2009, the English Department of Health had plans to double the number of adult intensive care beds available in an effort to mitigate the worst effects [3]; some of this additional capacity could be achieved by delaying elective surgeries, which itself could create additional health problems. Fortunately, the autumn/winter wave of the influenza pandemic was sufficiently mild that such action did not need to be taken, but it exemplifies the impact that future epidemics may have on hospitals.

School closures have been proposed as a viable method of reducing both the final size and peak of an epidemic [4]. Data from a variety of previous epidemics suggest that a significant proportion of seasonal influenza transmission occurs within schools, and that closing schools may reduce the peak by as much as 45 per cent [5]. Given that the 2009 H1N1 pandemic strain was more prevalent in children than usually observed for seasonal influenza, there was the possibility of an even greater reduction. This suggestion is supported by data from England: during June and early July, when schools were open, reported influenza cases were doubling approximately every week, however, during the school holidays estimated cases dropped by around 40 per cent each week [2]. Although these data are influenced by reporting biases and the impact of family vacations, they indicate the potential power of nation-wide long-term school closures. However, such closures have severe implications both economically and in terms of public-health services owing to the number of working parents (especially health-care workers) who would be forced to take time off work. For these reasons, and owing to the relatively mild nature of the infection, national school closures were not used in the 2009 outbreak, however in future outbreaks, small-scale reactive closures could be considered as a means of reducing the peak incidence in regions where hospitals are reaching capacity. It is this reactive use of school closures that we investigate here. Other causes of school closure during a pandemic, for example, absence of staff owing to illness or coincidence of the pandemic peak with bad weather, may play a similar epidemiological role to reactive school closure, although the timing of these events cannot be optimized by planning. When considering hospital capacity, we initially focus on adult ICU facilities for two reasons: first, ICU bed space is likely to be closer to capacity than standard hospital beds; and second, although paediatric ICUs are more sparsely distributed than adult ICUs, the existing National Health Service (NHS) strategy already has provision for either long-distance transfers or the use of adult ICUs for children as appropriate [3].

In this paper, we initially consider the probable reduction in peak cases (and therefore the probable reduction in peak demand on ICU facilities) that can be achieved by short-duration school closures. Next, using detailed socio-geographical information we consider the probable catchment area for each hospital and hence the relative burden on each ICU bed. Combining these two results allows us to predict the impact of localized school closures around those hospitals where demand for adult ICU beds exceeds capacity. This impact is measured both in terms of the number of hospitals where ICU demand exceeds capacity and the total distance that individuals need to be transported to the nearest ICU with spare capacity. Finally, a similar calculation is made for paediatric ICUs.

## 2. Methods

### (a) Epidemiological consequences of short-duration school closures

To gauge the approximate reduction in the epidemic peak that can be achieved through school closures, we use a standard age-structured epidemic model, with between age-group mixing derived from the pan-European POLYMOD survey [6,7] and described in detail in [8]. This model provides a good description of the dynamics of the 2009 pandemic in England; capturing the growth rates during school terms, the decline in cases during the school holidays and the overall magnitude of the epidemic.

Using the methods detailed in Baguelin *et al.* [8], we formulate a set of 600 age-dependent mixing matrices and age-dependent susceptibility profiles that are consistent with both the POLYMOD survey results and the observed 2009 pandemic in England. Using each of these parametrizations, we determine the peak epidemic incidence across all age-classes in the worst-case scenario when schools are open for the entire duration of the epidemic; we then determine the optimal timing (*t*_opt_) of a fixed duration school closure in terms of minimizing the peak incidence. For school closures enacted within half a day of this optimum (*t*_opt_ ± 1/2, thereby capturing the fact that schools will close for whole days), we calculate the relative peak incidence in children (under 15 years of age), adults (15–64 years old) and the elderly (over 65) compared with the peak without any intervention.

Given the range of uncertainty in many of the fundamental parameters, especially when considering future pandemics, we take a parsimonious approach and allow some of the population-scale emergent quantities to be parameters of our system. In particular, we take the peak national demand on adult ICU facilities (relative to the available capacity) and the reduction in this demand owing to well-implemented school closures to be two static parameters of our hospital-scale model.

### (b) Catchment area of each hospital and school

To estimate the burden that will be placed on each hospital, we first need to determine which hospital will be the preferred destination of adults who are taken ill. (The policy regarding children is different and discussed below.) We make the simplest assumption that all adults within a lower super output area (LSOA, the smallest spatial scale at which all necessary data are available, defined by the Office for National Statistics at www.ons.gov.uk) will attend the same hospital, and that the choice of hospital is a function of the (Euclidean) distance to the hospital. Throughout the main paper all results are formulated assuming that patients attend their nearest hospital, however, an alternative assumption involving the number of beds is considered in the electronic supplementary material.

**Figure 1.**
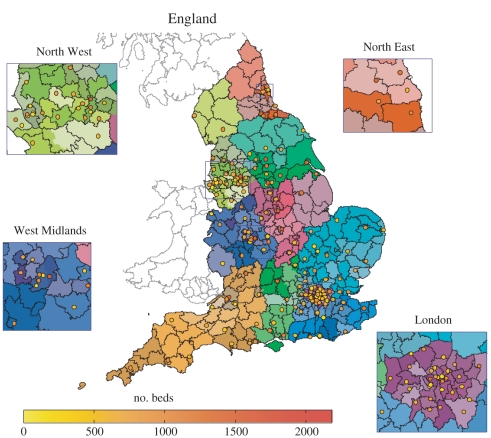
Different administrative regions and hosptial locations in England. Local authorities (LAs) are shown using grey lines for Scotland and Wales, and black lines for England. Strategic health authorities (SHAs) are shown using similar hues, and within these, primary care trusts (PCTs) are shown by different hue intensities and saturations. Acute trusts are shown by a circle at the location of the main hospital, with red indicating a larger number of total beds and yellow indicating a smaller number of total beds, as shown in the colourbar.

Figure 1 focuses on the overlapping geography associated with local administration of schools, primary care and hospitals in England. Hospitals are shown as points, colour-coded by their capacity; as expected, hospitals are aggregated in regions of high population density. In this figure, delineated regions correspond to local authorities (LAs) who have responsibility for local schools, while coloured regions correspond to strategic health authorities (SHAs) and within these, primary care trusts (PCT) who are responsible for local primary health care (while PCTs are not directly involved in our analysis, these bodies play a key role in the English NHS). The relationship between administrative units is not straightforward, although in the majority of cases each PCT can be seen to contain multiple LAs (with Birmingham a key exception).

**Figure 2.**
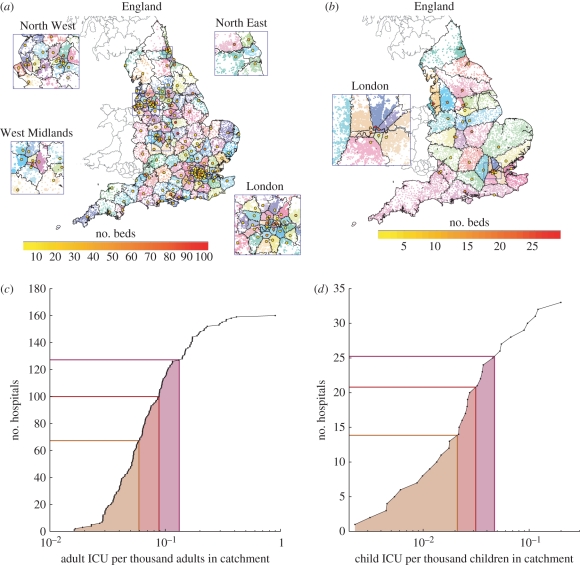
(*a,b*) Local authorities are shown using grey lines for Scotland and Wales, and black lines for England. English NHS trusts with (*a*) non-zero adult ICU capacity and (*b*) non-zero paediatric ICU capacity is shown by a circle at the location of the main hospital, with red indicating a larger number of ICU beds and yellow indicating a smaller number of ICU beds, as shown in the colourbars. The assumed catchment areas for these hospitals are shown using black lines containing coloured dots located at the population-weighted centroids of lower super output areas (LSOAs) to indicate population density. (*c*,*d*) The heterogeneity in local ICU capacity in these catchments for (*c*) adults and (*d*) children is also shown. The impact of local demand at a level consistent with certain levels of national demand is shown using coloured lines. *y*-axis intercepts give the number of hospitals locally over capacity at a given national level of demand. (*c*,*d*) Circles with solid line, cumulative distribution; orange line, 67% of national capacity; red line, 100% of national capacity; pink line, 150% of national capacity.

Catchment areas for each hospital, based on shortest distance (shown as delineated regions in figure 2*a*,*b*) cannot be simply related to either of the two administrative units shown in figure 1. The spatial arrangement of hospitals, together with assumed assignment to the closest hospital, leads to considerable heterogeneity in the number of individuals within the catchment area relative to the number of general or ICU beds, with no clear spatial pattern to this variability. Furthermore, even the use of alternative localized methods of assigning patients to hospitals that take into account the total number of beds do not substantially diminish this heterogeneity (see the electronic supplementary material).

Since paediatric ICU facilities are sparser and more geographically heterogeneous, the issue of local overcapacity is posed in a different way. While we assume that individuals needing this specialized form of care are optimally assigned to the nearest national unit that is not overcapacity, there is still a local question of hospitals being unable to offer paediatric ICU to individuals closest to them, leading to excessive journey times and posing the question of whether localized school closures would be worthwhile.

To determine the catchment area of a school, we use data on pupils’ home locations at an LSOA level, collected and provided to us by the Department for Children, Schools and Families, and so do not need to make distance-based allocations. The typical form of these data is shown in the electronic supplementary material.

### (c) Implementing local school closures

For each hospital where the peak demand on adult ICU beds is above capacity we consider local closure of schools. Throughout the paper we assume that schools can be shut independently, and also consider the alternative that school closures are coordinated through the LA leading to all schools under the control of an LA closing simultaneously. We make the optimal assumption that closures are implemented sequentially in the order that maximizes the impact on the associated hospital; in practice, this means first implementing closures of schools (individually or in LAs) that have the greatest population overlap between school and hospital catchment areas.

The impact that closing schools has on the peak demand for each hospital is assumed to scale linearly with the proportion of hospital catchment area affected. For example, if 40 per cent of school children in a hospital catchment area are away from school owing to local closures, then the reduction in peak demand on the hospital is assumed to be 40 per cent of the maximum (the validity of this assumption is tested in the electronic supplementary material). As such, each school closure may reduce the demand on multiple hospitals, owing to the complex overlap between the catchment areas of each.

### (d) Moving patients to alternative ICUs

To determine the total distances moved between adult patients’ home locations and the ICU they are treated at, we use a Monte Carlo algorithm in which patients are randomly permuted and assigned to the available ICU bed nearest to their local hospital (which can simply be at their local hospital if it remains under capacity).

## 3. Results

**Figure 3.**
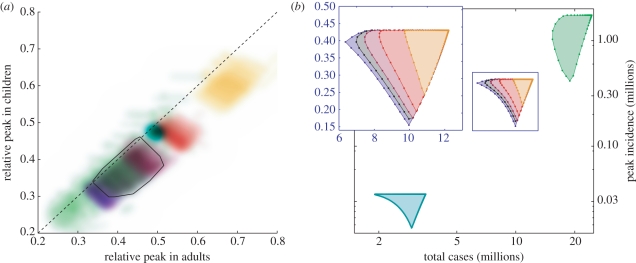
Impact of duration of school closure and epidemiological assumptions on the peak incidence in adults and children relative to simulations without school closures. (*a*) For each colour, there are 6000 points reflecting uncertainty in the mixing matrix and imperfection in the timing of closures as they would have to coincide with whole days. The default model has *R*_0_ = 1.4 and a single continuous closure of four weeks; for clarity, the convex hull around the default points is shown as a solid black line. Alternative values of *R*_0_ are shown in turquoise (*R*_0_ = 1.1) and green (*R*_0_ = 2.0). Orange, crimson and dark red points indicate shorter continuous closures of one, two and three weeks; while purple represents an optimally timed strategy of two closures of two weeks each. (*b*) Impact of different timings of closure at the most probable parameter values. Dots are spaced a day apart, with no intervention at the extreme top right of each polygon, and motion clockwise around each polygon representing later closures. Note that the inset has linear axes while the main plot has logarithmic axes. In comparing the two panels, the minimum peak incidence for a coloured polygon in (*b*) divided by the maximum peak incidence on that polygon is a number comparable to the location of the corresponding coloured region in (*a*). (*a*,*b*) Black circles, default; yellow circles, closure = 7 days; red circles, closure = 14 days; maroon circles, closure = 21 days; purple circles, two closure; blue circles, *R*_0_ = 1.1; green circles, *R*_0_ = 2.0.

We simulate the impact of near optimally timed school closures lasting from between one and four weeks, and for different basic reproductive ratios (*R*_0_ = 1.1, 1.4, 2.0), and measure the relative peak incidence in children, adults and the elderly for all 600 plausible parametrizations. The results for children and adults are plotted in figure 3; results for the elderly resemble those for adults. These relatively short-duration school closures reduce the peak incidence by between 30 and 70 per cent, depending on both the duration and underlying epidemiological assumptions. Many results emerge from these simulations: first, as expected, a greater reduction in the peak can be achieved by either longer duration school closures or multiple closures of the same total duration. The corollary of this (which follows logically but is not explicitly modelled here) is that infections with a shorter generation time (shorter latent or infectious period) are more affected by school closures, as a closure of a fixed duration covers more generations of infection. Second, higher values of *R*_0_ lead to a greater impact for school closures, although the results are much more variable. In addition, the impact of school closures is generally greater on the peak incidence in children than in adults. Finally, it should be noted that we have been extremely optimistic in our timing of school closures, first in assuming that the optimum can be precisely calculated and second in assuming that school closures occur as close as possible to this optimum, although conditional on schools closing for whole days. If, for practical reasons, schools close for whole weeks (e.g. first closing on a Monday), then clearly there would often be a greater disparity between optimal and actual timings of the closure, reducing the impact on the epidemic peak. Surprisingly, the impact of alternative susceptibility profiles, such as those associated with seasonal influenza or the 1918 pandemic, is relatively minor.

Figure 2 shows the relative distribution of peak demand per ICU bed across all hospitals (assuming all regions experience identical peak levels of infection). We stress that the shape of this distribution is invariant under different epidemic profiles, it is merely that the absolute demand scales with the epidemic peak; in addition we find that there is generally insufficient age-structured heterogeneity between hospital catchment areas for these results to be strongly influenced by age-dependent severity of infection (see the electronic supplementary material). These results suggest that even if there is sufficient national capacity to deal with the peak demand during an outbreak, many hospital ICUs could be overwhelmed—an effect, which could potentially be exacerbated if some regions experience substantially higher epidemic peaks than the average.

**Figure 4.**
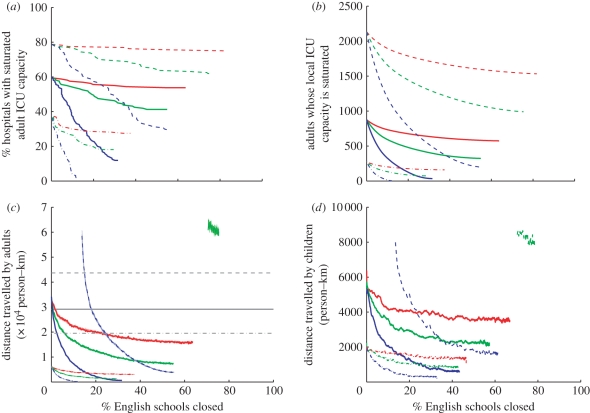
Effect of school closures on pressure on capacity as a function of the percentage of schools closed. Red lines represent the scenario where school closures can reduce the local peak by 15%, green lines 30% and blue lines 60%. From top to bottom, in each graph, the series of lines represent different levels of national peak adult ICU demand as a percentage of adult ICU demand relative to national capacity: 150% (dashed line), 100% (solid line) and 67% (dashed-dotted line). No red dashed line is shown in (*c*,*d*) because no amount of travel satisfies ICU demand in this scenario. (*a*) Percentage of hospitals whose capacity is saturated, (*b*) number of adult patients whose local hospital's ICU capacity has been saturated, and (*c*) total distance travelled by adults to their nearest hospitals (in grey) and the secondary distance travelled to a hospital with available ICU beds (mean as a dark-coloured line on top of fainter lines for each realization). (*d*) Smoothed means for 10^3^ realizations of total distance travelled by children, who do not make a primary journey, but are allocated an under-capacity ICU through national coordination. The absence of a red line (and the truncation of green and blue lines) in the upper region of (*c*,*d*) represents the absence of any allocation strategy that will satisfy all demand for ICU.

Faced with this potential lack of capacity in some hospitals, we consider how closing schools and thereby reducing the local epidemic peak could be used to reduce the number of ICUs with excess demand, and we assess the scale at which such closures would need to be enacted. Three measures are used to capture the impact of these closures (figure 4): the percentage of hospitals where adult ICU demand from the local catchment area still exceeds capacity (figure 4*a*); the maximum number of adults requiring ICU above local capacity (figure 4*b*); and the total additional distance travelled if adults are moved from their primary hospital to the nearest secondary hospital with spare ICU capacity (figure 4*c*). Figure 4*d* shows the impact of specially targeted school closures on the equivalent distance travelled by children.

**Figure 5.**
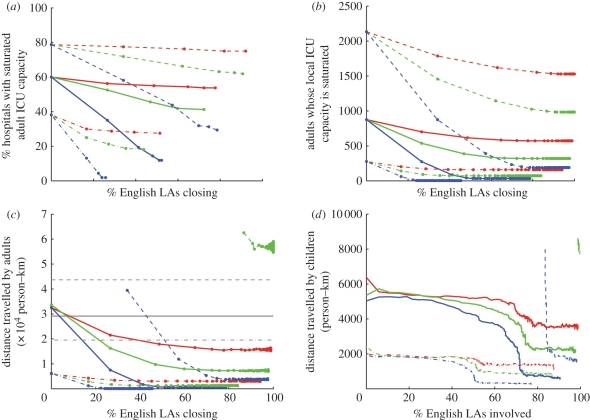
Effect of school closures on pressure on capacity as a function of the percentage of LAs closing schools. Red lines represent the scenario where school closures can reduce the local peak by 15%, green lines 30% and blue lines 60%. From top to bottom, in each graph, the series of lines represent different levels of national peak adult ICU demand as a percentage of adult ICU demand relative to national capacity: 150% (dashed line), 100% (solid line) and 67% (dashed-dotted line). No red dashed line is shown in (*c*,*d*) because no amount of travel satisfies ICU demand in this scenario. (*a*) Percentage of hospitals whose capacity is saturated, (*b*) number of adult patients whose local hospital's ICU capacity has been saturated and, (*c*) total distance travelled by adults to their nearest hospitals (in grey) and the secondary distance travelled to a hospital with available ICU beds (mean as a dark-coloured line on top of fainter lines for each realization). (*d*) Smoothed means for 10^3^ realizations of total distance travelled by children, who do not make a primary journey, but are allocated an under-capacity ICU through national coordination. The absence of a red line (and the truncation of green and blue lines) in the upper region of (*c*,*d*) represents the absence of any allocation strategy that will satisfy all demand for ICU.

Similar results are given in figure 5, for the situation when all schools under the control of an LA are closed simultaneously; obviously, this gives less benefit per school closure as there is less control over the targeting of closures, and significantly less benefit for the case of paediatric ICU.

Considering the scenario where, in the absence of school closures, peak national demand equals national capacity (solid lines in figure 4) we observe that 60 per cent of hospitals have a local demand that exceeds their capacity. Even with broadly optimistic assumptions about school closures (reducing the peak demand by 60%, blue line), the proportion of hospitals above capacity cannot be brought to zero and only achieves its lowest value of 12 per cent when there is a coordinated closure of at least 30 per cent of all English schools. While this coordinated closure still leaves 12 per cent of hospitals above capacity it does substantially reduce the amount by which the capacity is exceeded in these regions and, therefore, also reduces the distance patients needing ICU facilities have to be moved. Alternative, less optimistic assumptions concerning school closures (green and red lines) correspondingly have a more limited impact. Also shown in figure 4 are corresponding results for more-severe (dashed lines) and less-severe (dot-dashed lines) epidemics, when peak demand reaches 150 and 67 per cent of national capacity, respectively. Although the public-health consequences of different epidemic severity are marked, the relative impact of localized school closures is remarkably consistent.

## 4. Discussion

The decision to close schools clearly involves a trade-off between a variety of conflicting factors. Epidemiologically, social distancing measures such as school closures can reduce the peak height of an epidemic and therefore provide a benefit to hospitals; however they also have substantial economic and social impacts [4,9,10], and place additional pressures on business and health-care services that may already be struggling with absenteeism owing to illness. The large number of health-care workers with responsibilities for children of school age could result in the rate of absenteeism owing to school closure exceeding 30 per cent in hospitals and other health-care settings [9]. In a situation, where the potential capacity benefits of staff redeployment are already fully realized, this absenteeism rate would effectively discount the first 30 per cent of benefits in peak reduction owing to school closure.

In this paper we have made broadly optimistic assumptions about the impact of localized short-duration school closures. Two potential difficulties exist with the use of these methods. The first is that a large number of schools must close in a coordinated way if there is to be a substantial impact on the peak epidemic size. It is probably that individual schools may need to close owing to the impact of the epidemic on staff availability, however, these closures are unlikely to be sufficiently coordinated to offer protection to the surrounding population. Second, such closures must be carefully timed to start just before the expected epidemic peak, prediction of this optimal timing requires well-parametrized mathematical models that can account for the true number of cases and the pre-existing levels of susceptibility within the population. This is exceedingly difficult, as the nonlinear growth rate which is the key to determine the susceptible depletion becomes the only significant factor as the epidemic approaches its peak.

Our results are based on the English spatial pattern of hospitals, schools and population density, but we expect our conclusions to be widely applicable to other developed countries owing to the general difference in scales between hospital and school catchment areas and the ubiquitous heterogeneity in population density and hospital capacity. Precise predictions of hospital demand and the impact of school closures for other countries will be critically dependent on public-health measures and a number of social factors; however, our conclusions hold for a wide range of parameter assumptions and conclusions are therefore likely to be robust.

Given these results we conclude that there are significant limits on the scenarios for which a policy of school closures would be advantageous. When the predicted peak demand is likely to exceed national capacity (see dashed lines in figure 4), then a coordinated and possibly extended period of school closures may be necessary. Second, for some hospitals where demand exceeds capacity, owing to geographical features, there may be a close correspondence between school and hospital catchment areas; in such situations the closure of local schools is likely to have a very targeted impact on the peak demand. However, even in these cases, there are still the problems of economic and social impacts, and the difficulties of predicting the optimal timing of closures to be overcome.
